# Role of Air Pollution and rs10830963 Polymorphism on the Incidence of Type 2 Diabetes: Tehran Cardiometabolic Genetic Study

**DOI:** 10.1155/2020/2928618

**Published:** 2020-09-07

**Authors:** Fatemeh Jabbari, Anoushiravan Mohseni Bandpei, Maryam S. Daneshpour, Abbas Shahsavani, Seyed Saeed Hashemi Nazari, Hassanali Faraji Sabokbar, Amir abbas Momenan, Fereidoun Azizi

**Affiliations:** ^1^Department of Environmental Health Engineering, School of Public Health and Safety, Shahid Beheshti University of Medical Sciences, Tehran, Iran; ^2^Environmental and Occupational Hazards Control Research Center, Shahid Beheshti University of Medical Sciences, Tehran, Iran; ^3^Cellular and Molecular Endocrine Research Center, Research Institute for Endocrine Sciences, Shahid Beheshti University of Medical Sciences, Tehran, Iran; ^4^Prevention of Cardiovascular Disease Research Center, Department of Epidemiology, School of Public Health and Safety, Shahid Beheshti University of Medical Science, Tehran, Iran; ^5^Department of Human Geography, Tehran University of Sciences, Tehran, Iran; ^6^Prevention of Metabolic Disorders Research Center, Research Institute for Endocrine Sciences, Shahid Beheshti University of Medical Sciences, Tehran, Iran; ^7^Endocrine Research Center, Research Institute for Endocrine Sciences, Shahid Beheshti University of Medical Sciences, Tehran, Iran

## Abstract

Diabetes mellitus (DM) is considered one of the leading health issues that are egregiously threatening human life throughout the world. Several epidemiological studies have examined the relationship of a particular matter < 10 *μ*m (PM10) exposure and with type 2 diabetes mellitus (T2DM) prevalence and incidence. Accordingly, the current study is a study investigating the independent influence of air pollution (AP) and rs10830963 on the incidence of T2DM. A total number of 2428 adults over 20 years of age participated in a prospective cohort (TCGS) during a 9-year follow-up phase. The concentration of AP was measured, and the obtained values were considered the mean level in three previous years since the exposure concentration took the people living in that location. The COX regression model was employed to determine the influence of AP and rs10830963 on the incidence of T2DM in adjustment with covariate factors. Among the 392 T2DM, 230 cases (58.7%) were female diabetics, and 162 (41.3%) were male diabetics. According to the multivariable-adjusted model, exposure to PM10 (per 10 *μ*m/m3), associated with the risk of T2DM, although just a borderline (*p* = 0.07) was found in the multivariable model (HR; 1.50, 95% CI; 1-2.32). The rs10830963 was directly associated with the incidence of diabetes, and the GG genotype increased the T2DM rate by 113% (more than two times) (HR; 2.134, 95% CI; 1.42-3.21, *p* ≤ 0.001) and GC increased it by 65% (HR; 1.65, 95% CI; 1.24-2.21, *p* ≤ 0.001). Long-term exposure to PM10 was associated with an increased risk of diabetes. Thus, it is suggested that the individuals with variant rs10830963 genotypes fall within a group susceptible to an increased risk of T2DM arising from AP.

## 1. Introduction

Today, diabetes mellitus (DM) is considered one among the main health issues that are egregiously threatening human life throughout the world [1]. DM is the seventh cause of death around the globe, and its prevalence is rising [2]. In this regard, the International Diabetes Federation (IDF) has predicted that there have been at least 415 million diabetes sufferers within the age range of 20–79 years in 2015, and it has been estimated that this number will amount to 642 million cases in 2040 [3]. A variety of factors, including genetics [4, 5], lack of physical activity, increased body mass index (BMI), smoking, bad dietary habits, and exposure to AP are among the factors leading to the emergence of type 2 diabetes mellitus (T2DM) [5, 6]. In recent years, AP has been at play as a critical issue that has had a terrible toxicological effect on human beings and the environment [7]. In this vein, 5.25% of all deaths have arisen from ambient particulate matter pollution. It is noteworthy that above 80% of human beings are threatened directly by AP in the world, which has strongly exceeded the allowed limit of the World Health Organization (WHO) [8]. In terms of AP, Iran stands the third top country in the world, and this has resulted in substantial financial losses each year, i.e., 16 billion dollars. Indeed, there are only four cities in Iran that bring about the bulk of AP in such a way that only Tehran has resulted in the number of 4460 deaths in 2013, while the real number seemed to be more severe and is getting worse annually [7].

Taking a look at the related literature, one may come with several epidemiological studies that have examined the relationship of particular matter (PM10, an essential component of AP) exposure with T2DM prevalence and incidence [8, 9]. These studies have yielded different research findings, and no consistent results in this regard are available. Some of them have observed an indirect relationship between the variables as mentioned earlier [10, 11], while some others have not [12]. Another factor that is likely to affect one's response to this disease when encountering environmental events is genetic background [5]. There is an interaction between gene activity and the environment, so these diabetic-related pathways are influenced by air pollution [13]. Besides, researchers investigated that gene-environment interactions are a potential factor for the modification of genetic variants and increase the risks of diabetes [14–16]. In the same way, Eze et al. investigated the genetic risk score of the people with diabetes exposed to AP (particulate matter) concerning 63 genetic variants for a 10-year period, as the genetic markers, which are specifically associated with the heightened risk of T2DM. Their results showed that five single variants, namely, GRB14, UBE2E2, PTPRD, VPS26A, and KCNQ1 had a nominally significant interaction with PM10 [13]. Another study conducted by Eze et al. revealed that IL6-572G > C and IL6-174G > C and PM10 did not have any association with each other; however, PM10 and T2DM had a significant positive association with each other [17]. Recently, diabetes has witnessed an increasing trend in a nonstop manner in all countries of the world, i.e., both developed and developing countries. In this regard, despite the deluge of studies being carried out in developed countries, the relevant studies in developing and underdeveloped countries are scarce, thereby, it is required to undertake research in this domain in such countries [9]. Furthermore, only a limited number of epidemiological studies have examined the interactive impact of AP and SNP on the risks of T2DM. Accordingly, the current study is a longitudinal one that is aimed at investigating the independent influence of AP and SNP on the incidence of T2DM. It was attempted to further explore the interactive outcomes of the exposure to AP with the selected SNP in order to determine the association between AP and T2DM.

## 2. Methods

### 2.1. Study Population

Tehran Lipid and Glucose Study (TLGS) is an open-ended prospective population-based cohort project of a representative sample of dwellers in Tehran (the capital city of Iran), in the eligible age (≥3 years) at the time of recruitment. In brief, this is a cohort study that was initiated in municipal district 13 of Tehran [18, 19].

Tehran cardiometabolic genetic study (TCGS) is a family survey of participants in the 20-year TLGS to determine the prevalence of risk factors for noncommunicable diseases, such as metabolic syndrome. Moreover, it is aimed at assessing the impact of a healthy lifestyle on improving risk factors and preventing the growing trend of noncommunicable diseases, including type 2 diabetes and serum lipid disorders. This project has begun since 1999 and is still going on. The genomic study of participants in the TCGS study was carried out in a family context whose details have been described in [20].

**Figure 1:**
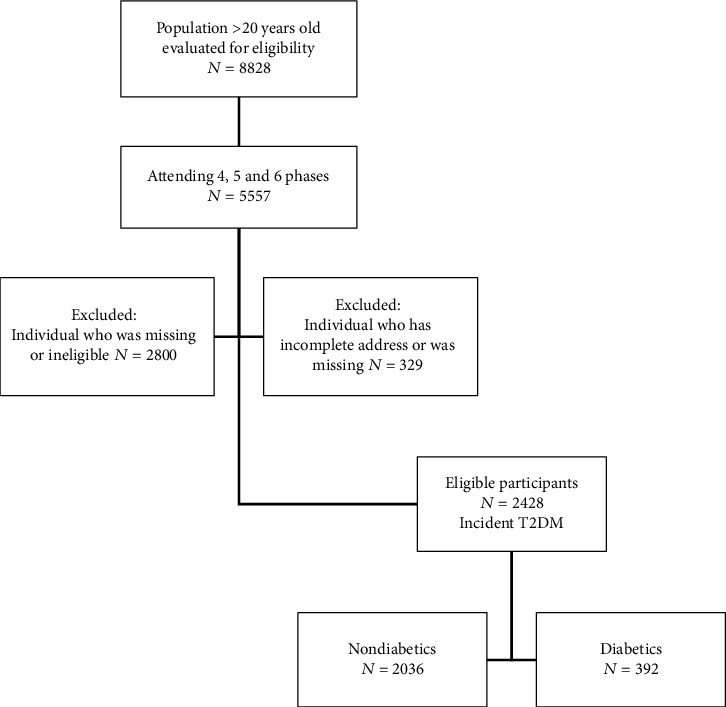
Flow diagram of the study design and participants in the TCGS cohort.

In the present study, considering the availability of data on AP, the participants in phases 4 to 6 of the TCGS cohort were evaluated. In this way, phase 4 was considered the baseline and, then, both healthy individuals over 20 years of age (*n* = 5557). It is noteworthy that 2236 participants who had diabetes or prediabetic at baseline (phase 4) were excluded; thus, new diabetic cases were selected for each phase. Also, only 564 cases had not completed information to be selected as cases or control and hence were considered missing data. Three hundred twenty-nine individuals with incomplete address (required for geocoding) or missing addresses were excluded from the study, and eventually, 2428 cases were selected and enrolled (Figure 1).

### 2.2. Definitions

In this study, type 2 diabetes mellitus was defined as the concentration of FBG ≥ 126 mg/dL, non − FBG ≥ 200 mg/dL, and regular use of glucose-lowering medication.

### 2.3. Exposure Assessment

The data about ambient AP were received from 21 Tehran Air Quality Control Company (TAQCC) within an interval of three years (2009–2011) at air monitoring stations in Iran. Then, the concentrations of PM10 (particulate matter < 10 *μ*m, *μ*g/m^3^) were evaluated continuously and recorded each hour in all stations. In the next analyses, the daily average concentration of PM10 (24 hours) was measured utilizing the available data, i.e., at least 75% of valid hourly numerical data for the days and the minimum 75% of valid daily values for the years. When the data of the average daily amounts of air pollutants were not at hand in one air monitoring station, the missing values were filled out by putting the average number of other stations and the same type of that day instead of the missing values, as proposed by Jung et al. [21].

**Figure 2:**
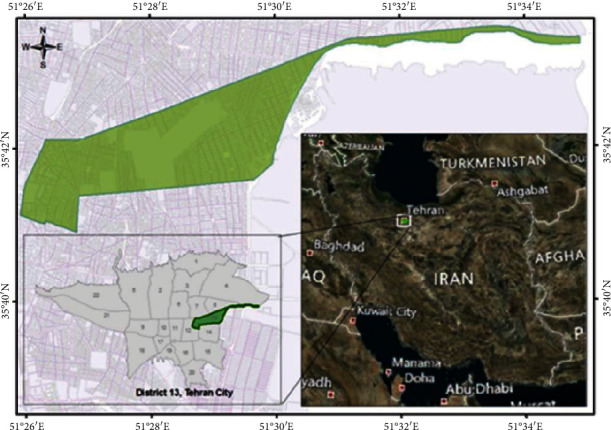
Location of the study area.

Here, to determine the specific locations of the air monitoring stations and obtain the data about air pollutants were benefited from the geographic information system (ArcGIS10; ESRI, USA). Furthermore, the inverse distance weighting (IDW) method was employed to measure the yearly concentration of air pollutants [21, 22]. Since this cohort study is being carried out in district 13 of Tehran (Figure 2.), this area was demarcated through the order “extract by mask” in GIS software. Then, all the 2428 participants' addresses were converted to geocoded residential addresses, and the annual concentration of PM10 data was extracted for each participant.

In the end, the concentration of each air pollutant was measured, and the obtained values were considered the mean concentration in three previous years since the exposure concentration took the people living in that location into account.

### 2.4. Covariates

Factors such as age, gender, BMI, smoking status, education level, income status, and physical activity were used as covariates. BMI was calculated as the weight (kg) divided by the square of height (m) [1, 23].

Smoking status recodes as never, former, and current smoker. Ever smokers were assigned into two groups: a participant who has smoked greater than 100 cigarettes in his/her lifetime and has smoked in the last 28 days was considered a current smoker, and ever smokers who have smoked greater than 100 cigarettes in their lifetime but have quitted smoking were considered former/ex-smoker [24].

The literacy level of the subjects was divided into three groups based on the number of years of schooling [25, 26], education (literate: <6 years, 6–12 years, or >12 years schooling). In terms of job and income, it is divided into two categories, occupy (yes or no) [27, 28], physical activity (two groups <500 or >500 Met-min/Week), systolic blood pressure and diastolic blood pressure (mmHg), and LDL and HDL (mg/dL) were considered the covariates in this research. It is noteworthy that the data, as mentioned earlier, are generally recorded at every stage of the cohort.

### 2.5. Genotyping

Here, genomic DNA was obtained from the buffy-coat of each sample using a proteinase K/salting out standard method. After that, some portions of the DNA samples got genotyped with HumanOmniExpress-24-v1-0 bead chips (containing 649,932 SNP loci with the average mean distance of 4 kb) at deCODE genetics company (Reykjavik, Iceland) based on the manufacturer's instructions (Illumina Inc., San Diego, CA, USA). Then, the data on genotyping polymorphisms were analyzed, and the rs10830963 was selected for the conduct of association analysis.

### 2.6. Statistical Analyses

Genetic R package V.5 was used to determine the Hardy–Weinberg equilibrium. In this regard, the COX regression model was employed to assess the influence of AP and SNP on the incidence of T2DM in adjustment with covariate factors. The Schoenfeld residual test was used to examine the proportional hazard assumption of COX models, and then, the proportionality was obtained. Time-in-study (i.e., follow-up time) was the timescale employed in the intended models where one crude model and three multivariable models got designed to make a comparison between the covariates' effects.

The results showed that Model 1 did not have any adjustment, Model 2 shows the effect of PM10 and SNP, Model 3 shows interaction of PM10 and SNP, Model 4 show the effect of PM10, SNP, age and gender, and Model 5 adjusted for all covariates (age, gender, BMI, SBP, DBP, SNP, education, smoking status, physical activity (Leisure_Met), occupy, LDL, and HDL cholesterol).

Accordingly, HR was considered the risk of the incidence of diabetes for PM10 with 95% CI. Moreover, the statistical analyses were conducted using STATA (v.14). In this step, 0.05 was considered the significance level for exposure and interaction effects. Here, the maximum missing values were considered for physical activity and education levels.

## 3. Results

A total number of 2428 adults over 20 years of age participated in this study during a 9-year follow-up phase. Among the 392 T2DM, 230 cases (58.7%) were female, and 162 (41.3%) were male. The characteristics of the participants have been shown in Tables 1 and 2. The results showed that the mean values of participants' age and BMI is equal to 45.4 (13.3) years and 28.09 (4.8), respectively.

It was shown that most of the T2DM cases, i.e., 172 cases (43.9%) were placed in a 50-to-69-year-old age group. The actual BMI of the majority of participants was ≥30 kg/m^2^ (49.2%). Besides, the incidence rate of 94.5% of T2DM took place after the age of 35 years.

The mean systolic blood pressure (SBP) and diastolic blood pressure (DBP) levels in diabetics were 125 (19.44) and 80.25 (12.1), respectively, which were higher than nondiabetics 112.8 (15.52) and 76 (10.53), respectively. The mean BMI in females with diabetes was 30.8 (5.1), which was higher than in male 27.33 (4.54). 81.1% of diabetics never smoked.

The mean, min, and max values of the AP levels at district 13 of Tehran in 2009-2011 have been shown in Table 2. As it has been shown, the mean, interquartile range (IQR) has been obtained 80-85 *μ*g/m^3^. In three years, PM10 levels have witnessed an approximate increase of 1.1 annually. The mean value of PM10 concentrations was 82.6 *μ*g/m^3^.

In this regard, rs10830963 (MTNR1BC > G) was below the Hardy–Weinberg equilibrium (HWE) (*p* > 0.05) and the frequency minor allele frequency (MAF) for G allele was 0.31.

A total of 52.5% participant was the G allele, compared to 47.5%, who was the C allele. The CC and GG+GC genotypes were, respectively, 39.5% and 60.5% in diabetics and were, respectively, 49% and 51% in nondiabetics.

In the current study, over 62.5% of the participants resided in high PM10 districts (ambient PM10 levels > 81 *μ*g/m^3^), where the mean values of their age and BMI were 44.6 (13.1) years and 26.7 (4.51), respectively.

After the application of a COX regression, one of the important findings in this study was the revelation of the significant risk of T2DM in the participants. According to the crude and multivariable-adjusted model, exposure to PM10 (per 10 *μ*m/m^3^) and the risk of T2DM were associated with each other, although just a borderline was found in the multivariable model (Table 3). Similarly, an increase of 43% and 50% per 10 *μ*g/m^3^ of PM10 in T2DM incidence was observed in the crude (HR; 1.43, 95% CI; 1-2.23) and multivariable-adjusted model (HR; 1.50, 95% CI; 1.003-2.32) in the current study.

Furthermore, a significant association was observed between the rs10830963 candidates and diabetics across the five models (Table 3). On the other hand, the interaction between PM10 exposure and rs10830963 was not associated with the incidence of T2DM (HR; 1.1, 95% CI; 0.67-1.80). Besides, rs10830963 and covariates in the multiple pollutant models (which included PM10) had an association with age, BMI, SBP, and HDL (*p* < 0.05); however, no other association was observed. In the same way, rs10830963 was directly associated with the incidence of diabetes and the GG genotype increased the T2DM incidence by 113% (more than two times ((HR; 2.134, 95% CI; 1.42-3.21) and GC increased it by 65% (HR; 1.65, 95% CI; 1.24-2.21).

## 4. Discussion

Indeed, this study was a longitudinal one that was conducted to examine the association between PM10 and rs10830963 on T2DM incidence. The pooled dataset consisted of 392 diabetic cases and 2036 normal individuals. The female participants outnumbered the male ones, and 57.6% of them held the GC or GG variant.

It was shown that traffic-related AP had a significant association with the T2DM incidence. The current PM10 estimates were obtained higher than the levels found in other studies (Table 3). The PM10 results obtained in this study are in line with the qualitative findings of some other cohort studies [6, 29].

A considerable complexity was observed in the major biological mechanisms by which PM10 exposure results in the appearance of diabetes. Prior studies have reported that the long-term exposure to PM10 was associated with a greater homeostatic model assessment of insulin resistance (HOMA-IR) and fasting insulin concentrations; therefore, it brings about an increased risk of diabetes [30].

Similarly, Hansen et al. [31] carried out a cohort study on nurses, and a positive association was observed between PM10 and diabetes incidence. Weinmayr et al. [32] also reported a positive association between PM10 and the incidence of type 2 diabetes among people without diabetes.

The genes pertaining to diabetes risk are supposed to affect *β*-cell function directly or indirectly through insulin resistance [33]. In this study, the development of T2DM was affected by MTNR1B polymorphism (rs10830963), especially while being exposed to PM10 (*p* < 0.05). However, there was no significant interaction between PM10 and rs10830963 on diabetes incidence (HR; 1.1, 95% CI; 0.67-1.80).

In the same way, Eze et al. [13] conducted a cohort study on 6329 Swiss adults to examine the interaction of AP and genetic risk score, and they reported that the GRS of 63 SNPs did not significantly predict the incidence of type 2 diabetes for MTNR1B polymorphism (rs10830963).

In the same line, another study was conducted on the genetic risk score (GRS) of 49 SNPs, and the results represented the availability of a significant positive association of GRS with the incidence of T2D and MTNR1BC > G, and it was reported that this association could be modified by age and obesity [34].

In this regard, Eze et al. undertook another study [17] and indicated that there was an interaction between PM10 and proinflammatory candidate genes, such as IL6-572G > C and IL6-174G >C, which is in agreement with the hypothesis claiming the significant effect of air pollutants on T2DM via inflammatory pathways.

Accordingly, they found no association between diabetes and AP among the individuals with GG genotype and attributed this finding to the differences in PM10 constituents available in different regions. In this study, 60.5% of the diabetic individuals in comparison with 952 healthy subjects (51%) had at least one risk of G allele, which further determines the role of this gene variant in diabetes incidence.

In this exploratory study, a statistically significant association was observed between (PM10, RS) and age as the main covariate (HR; 1.04, 95% CI; 1.03–1.04).

The current findings showed that the majority of participants were placed in the age range of 35 to 49 years (42.6%). In addition, 72.4% of the study population had a BMI above 25, while BMI increases the risk of diabetes by 10% at large.

In a review study carried out by Li et al. [9] and a meta-analysis done by Alderete et al., [35] it has also been mentioned that the individuals exposed to AP, with higher age and obesity are more prone to diabetes. On the other hand, 59.5% of the diabetics individuals with a BMI above 25 were carriers of the GC+GG genotype. It indicates the role of genetics and obesity in the incidence of diabetes.

These findings are consistent with those of the study conducted by Langenberg et al., where it was found that the genetic risk score is of higher importance than lifestyle interventions and environmental conditions in the incidence of diabetes [34].

In terms of pollution and cigarette smoking, it seems that the ambient AP was twice as much as the standard range, and 82.2% of the participants have no positive history of smoking. Therefore, the role of smoking was not statistically significant. In the same vein, Astell-Burt et al. [36] reported that AP had a stronger effect on nonsmokers than smokers. Furthermore, in a meta-analysis study conducted by Alderete et al. [35], it was revealed that AP had a more severe effect on nonsmokers with diabetes.

In this study, 22.4% of the participants had more than 12 years of education and underwent diabetes to a smaller extent than the ones with 6 to 12 years of education (60.6%). In addition, 22% of the participants with more than 12 years of education had a risk of G allele. It needs to be said that the remaining 17% of the participants had smaller than six years of education.

It is natural that the individuals holding a higher educational status are likely to enjoy a more desired economic situation and better jobs; therefore, these people may experience less exposure to AP. Moreover, those with a higher education level benefit from a better awareness of the disadvantages of AP, and thereby, they are more likely to assign more attention to the optimal management of health issues, which will contribute to the decreased risk of T2DM. This finding is consistent with that of a study done by Yang et al. [6].

The risk of T2DM in people is developed by such lifestyle causes as physical activity. In this domain, research findings have proposed that there are lower levels of outdoor physical activity in locations with more intense AP. Thus, it is argued that physical activity can act as a mediator in the relationship between AP and T2DM [9].

The present findings indicate that T2DM responses to long-term AP may be more tangible than physical activity, and the ones exercising in the polluted air to a larger extent can experience an increased risk of diabetes by 3%, although this relationship was not statistically significant.

In this line, AP has a causal relationship with traffic situation and emission levels in such a way that the increase of the traffic burden has an indirect relationship with the reduced physical activity. Accordingly, the reduction of physical activity has a negative association with insulin resistance and T2DM.

A number of studies have investigated the extent to which T2DM can be attributed to AP after the modification of physical activity level. A strong need for the conduct of further longitudinal research is felt in order to examine the mediating role of physical activity and other variables [9]. On the other hand, the genetic factor (G allele frequency) was the same for both groups (smaller than 500 and higher than 500) in this study.

This study benefits from a number of strengths. For example, the exposure level in the residential addresses of the whole cohort was calculated, and some individual characteristics were also obtained. In addition, to the best of the researchers' knowledge, this was the first study in Iran that focuses on investigating the association between exposure to PM10 and SNP on diabetes incidence.

On the other hand, this study had some limitations, as well. For instance, there was no information about PM10 (as an important risk factor for the disease) before 2009 at the researchers' disposal. Similarly, some data on physical activity and education were not available.

## 5. Conclusion

Long-term exposure to PM10 was associated with an increased risk of diabetes in this cohort group. Thus, it is suggested that the individuals with variant rs10830963 genotypes fall within a group susceptible to an increased risk of T2DM arising from AP. It also seems that the design of control measures and the implementation of clean air rules and regulations are among the best strategies for the prevention of diabetes.

## Figures and Tables

**Table 1:** Distribution of demographic variables of the study population.

Characteristics	Female, *N* = 1410	Male, *N* = 1018	Total, *N* = 2428
Age, mean (SD) (years)	44.7 (12.5)	46.34 (14.3)	45.4 (13.31)
Incident T2DM, *N* (%)	230 (16.3)	162 (15.9)	392 (16.1)
Body mass index, mean (SD) (kg/m^2^)	28.7 (5.1)	26.81 (4.2)	28.09 (4.8)
Blood pressure, mean (SD) (mmHg)			
SBP, mean (SD) (mmHg)	112.3 (16.6)	118.2 (16.53)	114.75 (16.8)
DBP, mean (SD) (mmHg)	74.77 (10.78)	79.3 (10.73)	76.67 (11)
LDL-C, (mg/dL)	125.14 (37.14)	124.42 (35.8)	124.84 (36.6)
HDL-C, (mg/dL)	51.76 (11.5)	43 (9.64)	48.09 (11.6)
Education level			
≤6 years (reference)	268 (19.7)	132 (13.2)	400 (17)
Between 7-12 years	815 (60)	619 (61.8)	1434 (60.6)
>12 years	277 (20.4)	251 (25)	528 (22.4)
Occupy, *N* (%)			
No	1165 (82.6)	222 (21.8)	1387 (57.1)
Yes	245 (17.4)	796 (78.2)	1041 (43.9)
Leisure_Met (Met-minutes/week)	712.2 (945.56)	852.4 (965.6)	773 (957)
Smoking status, *N* (%)			
Never	1365 (96.8)	630 (61.9)	1677 (82.4)
Former	14 (1.0)	130 (12.8)	113 (5.6)
Current	31 (2.2)	258 (25.3)	246 (12.1)

**Table 2:** Background characteristics of participants by diabetes status.

Characteristics/covariates	Diabetics, *N* = 392	Nondiabetics, *N* = 2036
Age, mean (SD) (years)	53.4 (13.3)	43.9 (12.8)
Gender		
Female	230 (58.7)	1180 (58)
Male	162 (41.3)	856 (42)
Body mass index, mean (SD), (kg/m^2^)	30.8 (5.12)	27.33 (4.54)
Smoking status, *N* (%)		
Never	318 (81.1)	1677 (82.4)
Former	31 (7.9)	113 (5.6)
Current	43 (11.0)	246 (12.1)
Body mass index (kg/m^2^)		
<18.5	0	27 (1.3)
18.5-24.9	43 (11.0)	600 (29.5)
25-29.9	156 (39.8)	897 (44.1)
≥30	193 (49.2)	509 (25.0)
Blood pressure, mean (SD) (mmHg)		
SBP	125 (19.44)	112.8 (15.52)
DBP	80.2 (12.1)	76 (10.53)
Education level		
≤6 years (reference)	115 (30.7)	285 (14.3)
Between 7 and 12 years	202 (54.0)	1232 (62.0)
>12 years	57 (15.2)	471 (23.7)
Occupy		
No	259 (66.1)	1128 (55.4)
Yes	133 (33.9)	908 (44.6)
Leisure_Met (Met-minutes/week)		
≤500	126 (48.5)	756 (51.0)
>500	134 (51.5)	736 (49.0)
rs10830963 (additive model)		
CC	140 (39.5)	915 (49.0)
CG	162 (45.8)	772 (41.3)
GG	52 (14.7)	180 (9.6)
HDL (SD), (mg/dL)	45.18 (10.62)	48.65 (11.7)
LDL (SD), (mg/dL)	137.81 (38.3)	122.34 (35.7)
PM10, mean (SD) (*μ*g/m^3^)	82. 5 (3)	82.6 (3.02)
PM10, min (*μ*g/m^3^)	76.35	76.35
PM10, max (*μ*g/m^3^)	88	87.4

**Table 3:** Multivariable adjusted risk of type 2 diabetes with PM10 and polymorphism.

Model	Variables	Hazard ratio (95% CI)	*p* value
Model 1^a^	PM10	1.43 (1-2.23)	0.036
Model 2^b^	PM10	1.43 (1.003-2.03)	0.048
	rs10830963 (reference)	1	—
	rs10830963 (CG)	1.35 (1.08-1.7)	0.009
	rs10830963 (GG)	1.91 (1.39-2.63)	0.001
Model 3^c^	PM10	1.33 (0.8-2.24)	0.28
	rs10830963 (reference)	1	—
	rs10830963 (CG)	0.64 (0.01-39.54)	0.83
	rs10830963 (GG)	0.43 (0.00-1651.1)	0.84
	Interaction	1.1 (0.67-1.80)	0.72
Model 4^d^	PM10	1.33 (0.8-2.24)	0.06
	rs10830963 (reference)	1	—
	rs10830963 (CG)	1.50 (1.16-1.83)	0.001
	rs10830963 (GG)	2 (1.43-2.71)	0.001
	Age	1.04 (1.03-1.05)	0.001
	Gender (male as reference)	1 (0.8-1.23)	0.94
Model 5^e^	PM10	1.50 (1.003-2.32)	0.072
	rs10830963 (reference)	1	—
	rs10830963 (CG)	1.65 (1.24-2.21)	0.001
	rs10830963 (GG)	2.134 (1.42-3.21)	0.001
	Age	1.03 (1.01-1.04)	0.001
	Gender (male as reference)	1 (0.67-1.49)	0.99
	BMI	1.10 (1.07-1.14)	0.001
	SBP	1.01 (1.00-1.03)	0.014
	DBP	1 (1.00-1.01)	0.30
	LDL cholesterol	0.99(0.99-1.003)	0.60
	HDL cholesterol	0.97 (0.96-0.98)	0.001
	Smoking status		
	Never smoker (reference)	1	—
	Former	0.67 (0.24-1.84)	0.44
	Current	0.88 (0.5-1.55)	0.65
	Education level		
	≤6 years (reference)	1	—
	Between 7 and 12 years	1.01 (0.71-1.45)	0.95
	>12 years	0.82 (0.51-1.31)	0.41
	Occupy	0.94 (0.64-1.40)	0.78
	Leisure_Met	1.03 (0.8-1.35)	0.8

^a^Crude model (PM10 alone). ^b^Model 2 (PM10 and SNP). ^c^Model 3 (interaction), ^d^Adjusted for age and gender. ^e^Adjusted for age, gender, education level, BMI, SBP, DBP, occupy, education level, smoking, Leisure_Met, and LDL and HDL cholesterol.

## Data Availability

The data used to support the findings of this study are available from the corresponding author upon request.

## Abbreviations

DM: Diabetes mellitus
T2DM: Type 2 diabetes mellitus
TCGS: Tehran cardiometabolic genetic study
PM10: Particular matter < 10 *μ*m
AP: Air pollution
SNP: Single nucleotide polymorphisms
IDF: International Diabetes Federation
WHO: World Health Organization
TAQCC: Tehran Air Quality Control Company
IDW: Inverse distance weighting
LDL: Low-density lipoprotein
HDL: High-density lipoprotein
BMI: Body mass index
SBP: Systolic blood pressure
DBP: Diastolic blood pressure
HWE: Hardy–Weinberg equilibrium
MAF: Minor allele frequency
GRS: Genetic risk score
IQR: Interquartile range.
